# Multiple phenotypic traits including developmental impairment in a Chinese family with infantile convulsion and choreoathetosis syndrome: a case study expanding the clinical spectrum of *prrt2*-related syndrome

**DOI:** 10.1186/s12887-025-06180-9

**Published:** 2025-10-06

**Authors:** Ruohao Wu, Xiaojuan Li, Zhanwen He, Zhe Meng, Liyang Liang, Wenting Tang

**Affiliations:** 1 Department of Children’s Neuro-Endocrinology, Sun Yat-Sen Memorial Hospital, Sun Yat-Sen University, Guangzhou, 510120 Guangdong China; 2 Children’s Medical Center, Sun Yat-Sen Memorial Hospital, Sun Yat-Sen University, Guangzhou Guangdong, 510120 China; 3 Department of Cellular and Molecular Diagnostics, Sun Yat-Sen Memorial Hospital, Sun Yat-Sen University, Guangzhou, 510120 Guangdong China; 4 Department of Research and Molecular Diagnostics, Sun Yat-Sen University Cancer Center, Sun Yat-Sen University, Guangzhou, 510060 Guangdong China

**Keywords:** Infantile convulsion and choreoathetosis syndrome, *PRRT2*, Incomplete penetrance, Global development delay, Growth retardation

## Abstract

**Background:**

Pathogenic heterozygous variants in the gene encoding proline-rich transmembrane protein 2 (*PRRT2*) have been recently identified as the major cause of familial infantile convulsion and choreoathetosis syndrome (OMIM#602,066), a spectrum of autosomal dominant paroxysmal neurological disorders, including self-limited infantile epilepsy (SeLIE) and infantile convulsion that can be isolated (IC) or associated with paroxysmal kinesigenic dyskinesia (PKD/IC). Incomplete penetrance of *PRRT2* variants and variable phenotypes without developmental impairment have been widely reported in previous studies of this syndrome, but no studies to date have documented global development delay (GDD) with growth retardation (GR) occurred in a family with multiple phenotypes of this syndrome.

**Case presentation:**

Here, using family-based whole-exome sequencing, we identified a pathogenic heterozygous *PRRT2* variant (NM_145239.3: c.718C > T, p.Arg240*) in a 3-generation Chinese family of infantile convulsion and choreoathetosis syndrome. The variant was detected in five family members, of which two (*pedigree* III.1 and III.3) were diagnosed with PKD/IC, one (*pedigree* III.2) presented uncontrolled generalized/focal seizures with GDD and GR; the GR of this patient was aggravated with the progression of the epileptic condition; she was then diagnosed with IC and developmental impairment, one (*pedigree* II.2) was diagnosed with SeLIE, and one (*pedigree* II.3) was phenotypically unaffected and recognized as an obligate carrier.

**Conclusions:**

In conclusion, we reported a *PRRT2*-related syndrome family harboring multiple phenotypic features, including uncontrolled seizures with developmental impairment, which may potentially expand *PRRT2*-related clinical spectrum. Moreover, our findings suggest that children with *PRRT2*-related seizures/convulsions, especially those who suffer from uncontrolled multiple seizure types, should be aware of potential risks of having developmental impairment aggravation and need timely and effective antiepileptic medications.

**Supplementary Information:**

The online version contains supplementary material available at 10.1186/s12887-025-06180-9.

## Background

Infantile convulsion and choreoathetosis syndrome (OMIM#602,066) is a clinically and genetically heterogeneous neurological syndrome combining a spectrum of seizure disorders [self-limited infantile epilepsy (SeLIE)/infantile convulsion (IC)] and/or paroxysmal kinesigenic dyskinesia (PKD)] [1]. SeLIE and IC are characterized by a cluster of seizures or convulsions, mostly presenting as generalized seizure form, in normal infants with normal neuro-electrophysiology and neuroimaging tests at a mean age of 6 months [2]. In most cases, SeLIE and IC can reach remission spontaneously within 2 years and have no impairment of neurodevelopment and growth [3]. PKD are characterized by recurrent and transient unilateral or bilateral involuntary limbic movements, including dystonia, athetosis, chorea, ballism or their combination, induced by sudden voluntary movements, stress or startle [4].

Previous studies have confirmed that heterozygous variants in gene encoding proline-rich transmembrane protein 2 (*PRRT2*) are the leading cause of infantile convulsion and choreoathetosis syndrome, accounting for 11 ~ 45% of sporadic cases and 69 ~ 100% of familial cases in more than 330 families with different ethnic backgrounds [1, 5–8]. Meanwhile, many reported familial cases having *PRRT2* heterozygous variants presented PKD with infantile seizure/convulsion (SeLIE or IC) (PKD/IC). Thus, some studies have considered familial infantile convulsion and choreoathetosis syndrome and *PRRT2*-related syndrome may be the same disorder [9]. However, later reports have further identified many novel phenotypes related to *PRRT2*-related syndrome, such as hemiplegic migraine [10], benign myoclonus of early infancy [11], febrile seizures [12] and episodic ataxia [10]. Given the high variability of these phenotypes related to this syndrome, it is obvious that *PRRT2*-related syndrome is a complex neurological disease with increased heterogenous phenotypes; new reports of patients with *PRRT2*-related syndrome could further expand the broad phenotypic spectrum related to this syndromic condition.

Herein, we reported a Chinese family with five individuals who carried a pathogenic variant in *PRRT2*. Detailed phenotypic and genotypic features were elaborately analyzed for those family members, we observed that this family harbored multiple phenotypic features, including uncontrolled seizures with developmental impairment (neurodevelopmental delays with severe growth restriction), which may be a novel phenotype in *PRRT2*-related disorders. This observation can potentially broaden the spectrum of phenotypes related to heterozygous variants in *PRRT2* gene and provide some clues to support the speculation that *PRRT2*-related syndrome can be one of genetic epileptic and developmental encephalopathies.

## Case presentation

### Phenotypes description

#### pedigree III.2

A Chinese female infant presenting recurrent afebrile seizures initially sought medical attention in our children’s medical center at the age of 5 months. Her seizures usually occurred after a period of stare (e.g., staring at an object for a short period of time), and approximately 3 times a week; each episode lasting less than 60 s. A seizure event occurred after she stared at a tinfoil on her right hand, and was captured by her mother using cellphone, which was recognized as a typical generalized tonic–clonic seizures (GTCSs) (Supplementary File 1 and Supplementary Video 1). When seizures terminated, the baby girl seemed very tried and felled asleep soon. Electroencephalogram (EEG) recorded some asynchronous slow waves discharged in her bilateral temporal, occipital and frontal regions after a GTCS attack. She had been treated initially for seizure attacks with levetiracetam (LEV) 50 mg/kg/d for several months, but there was aggravation in her seizure attacks and the GTCSs became more frequent (5 times per week) since the age of 1 year and 3 months. Then, LEV was withdrawn and the girl’s guardians refused any further antiepileptic drugs (AEDs) used in their baby girl.

**Fig.1:**
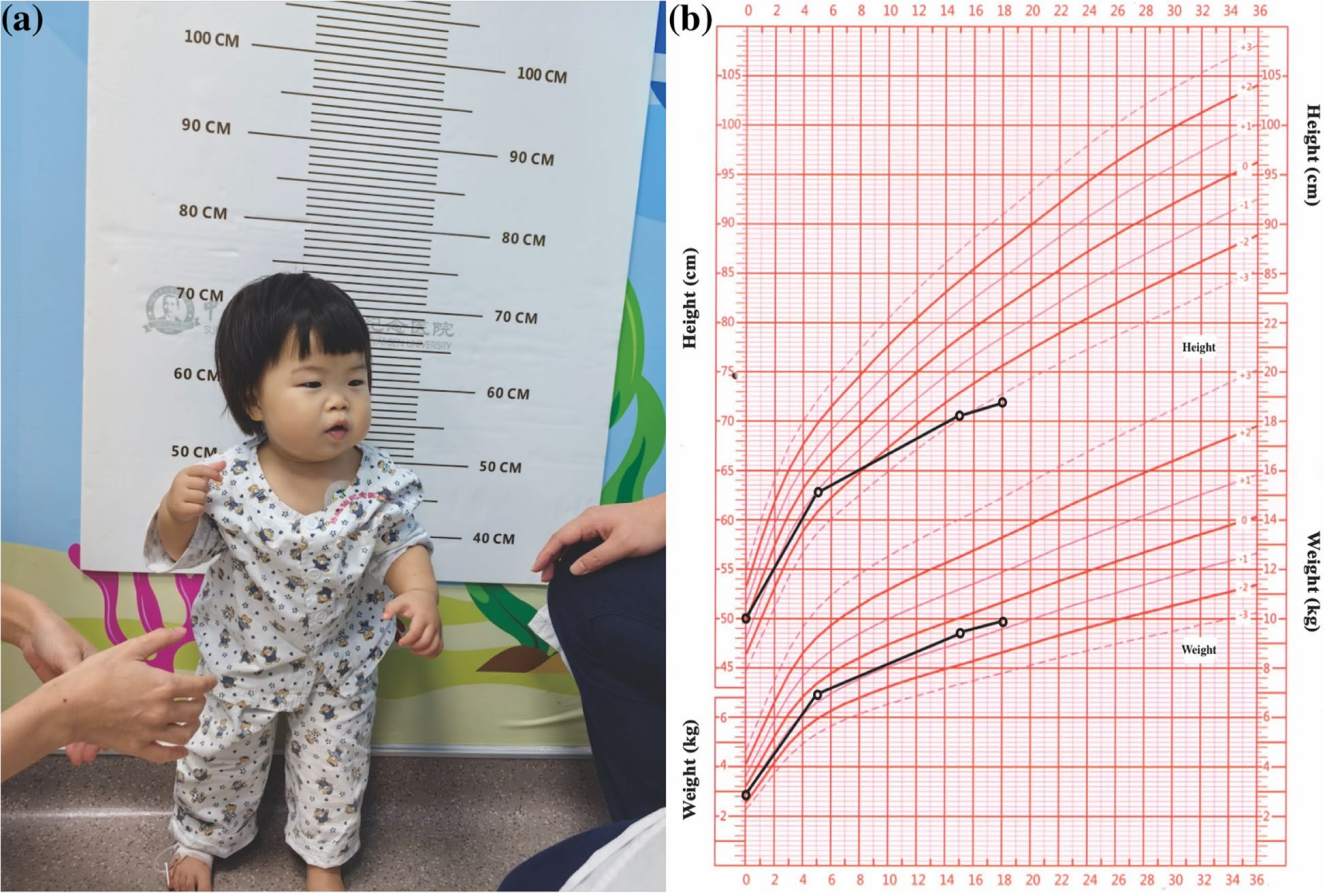
Clinical characteristics of the baby girl (*pedigree* III.2) in our children’s medical center. **a** Approximate height status of this girl at the age of 1 year and 8 months. **b** Her growth curve from birth to current age (1 year and 8 months)

At the age of 1 year and 3 months, she developed the focal seizures with impaired awareness triggered by staring at an object for a period of time, and was captured by her mother using cellphone (Supplementary File 1 and Supplementary Video 2). The duration of one focal seizure attack was mostly brief, typically lasting less than 30 s. It occurred approximately 1 time a week. Her parents still refused any antiepileptic treatments due to the fact that all convulsions or seizures in other family members (*pedigree* II.2, III.1 and III.3) who had experienced afebrile seizures were all self-limited; thus, the girl’ parents thought the condition of their baby girl could be also self-relieved regardless of the baby girl’s epileptic situation was still ongoing with no signs of clinical improvement until now. Cranial magnetic resonance imaging (MRI) showed normal brain structural images in this baby girl at the age of 1 year and 8 months (current age), but she had mild-moderate global developmental delay (GDD) with adaptation developmental quotient (DQ) score of 63, gross motor DQ score of 59, fine motor DQ score of 75, language DQ score of 59 and personal and social competence DQ score of 65. In addition, at the age of 1 year and 8 months her weight was approximately 10.5 kg (−0.8 *SD*), while her height was approximately 73.5 cm (< −3.0 *SD*) (Fig. 1a), indicating that she had severe growth retardation (GR). Importantly, the growth curve demonstrated that during the birth and the age of 5 months, the baby girl’s developmental levels of height were fluctuated between 0 ~ −1.0 *SD*; while after the age of 5 months since she developed recurrent afebrile GTCSs, her developmental levels of height were further decreased between −1.0 ~ −3.0 *SD*; when she developed multiple epileptic forms and her seizure attacks became more frequent since the age of 1 year and 3 months, her developmental levels of height were even fallen below −3.0 *SD* (Fig. 1b). All these findings indicate the potential positive association between the progression of epileptic condition and the severity of growth impairment in this patient.

#### pedigree III.3

The 8-year-old boy (the baby girl’s elder brother) had also experienced afebrile convulsions lasting up to 5 bouts of GTCSs in 3 months since the age of 4 months (Supplementary File 1 and Supplementary Video 3), however all seizures were self-limited and he had not required any AEDs. He had seizure-free without any antiepileptic treatments since the age of 8 months. Unlike *pedigree* III.2, he developed PKD triggered by startle at the age of 4 months. Each PKD episode lasting 30 to 50 s and all of them were accompanied by the bouts of GTCSs. The video demonstrated one attack episode that a series of paroxysmal choreiform limb movements mixing with left upper limb ballism in this boy; at that time, he was crying and seemed very upset and uncomfortable (Supplementary File 1 and Supplementary Video 4). Fortunately, there was no impairment of consciousness during the PKD attacks and a clinical self-remission was occurred when he got seizure-free at the age of 8 months. He also had normal neurodevelopment, growth, and cognition with unremarkable MRI brain imaging.

#### pedigree III.1

There was a similar medical history of infantile seizure in this female family member (the baby girl’s female cousin) with onset at that age of 6 months. However, she had only experienced a single bout of afebrile GTCSs with unremarkable EEG findings revealed by an urgent EEG testing. Besides, she also had normal neurodevelopment and growth. At the age of 17 years, she developed PKD triggered by stress or sudden movements, especially she initiated flipping her palm or sat suddenly. An attack episode was captured by herself and showed a typical PKD of left upper limb mixing with left lower limb when she suddenly flipped her left palm (Supplementary File 1 and Supplementary Video 5). Those attacks could be partially suppressed via tightening body muscle when she felt sensory aura, like paresthesia or sensations of limb muscle tension. Thus, the duration of those attacks was mostly brief (3 ~ 5 s), however the peak of frequency of those attacks was in her second decade (current age), and could reach several times in 1 day. Carbamazepine (CBZ) 0.2 g/d was then prescribed for her but she refused to use it because she could not tolerate the adverse drug reactions of CBZ, such as fatigue, nausea, headache, and skin rash.

#### pedigree II.2, and other family members

Similar to the *pedigree* III.1, her mother (*pedigree* II.2) also had experienced single bout of afebrile GTCSs at the age of 6 months, but she never had experienced PKD or PKD-like symptoms until now at the age of 40 years. A brief summary of phenotypic features of the 4 affected family members is demonstrated in Table 1. There was no similar history of infantile convulsion/epilepsy and PKD symptoms among other family members, including *pedigree* I.1, I.2, II.1, II.3 and II.4.

**Table 1:** Clinical characterics of the 4 affected individuals in the infantile convulsion and choreoathetosis syndrome family

			Features of SeLIE/IC						Features of PKD					
Individual pedigree ID	Sex	Age at present (y)	At onset (mo)	Seizure types	Seizure frequency	EEG	AEDs	Remission	At onset (y)	Trigger	Attack frequency	Sensory aura	Duration of attacks (s)	Remission
III.2	F	1.67	5	GTCS, Focal seizure	GTCS: 3 ~ 5 attacks per week Focal seizure: 1 attack per week	Asynchronous slow wave	Poor response to LEV	No	NA	NA	NA	NA	NA	NA
III.1	F	20	6	GTCS	Single until now	Normal	No	Yes	17	S/SM	Several times in 1 day	Yes	5 ~ 7	No
III.3	M	8	4	GTCS	5 attacks in 3 months	NA	No	Yes	0.33	S	5 times in 3 months	NA	30 ~ 60	Yes
II.2	F	40	6	GTCS	Single until now	NA	No	Yes	NA	NA	NA	NA	NA	NA

*IC* infantile convulsion, *SeLIE* self-limited infantile epilepsy, *PKD* paroxysmal kinesigenic dyskinesia, *y* year-old, *mo* month-old, *EEG* electronencephalogram, *AEDs* antiepileptic drugs, *F/M* female/male, *GTCS* generalized tonic-clonic seizure, *LEV* levetiracetam, *NA* none or not available; S, stress or startle; SM, sudden movement

### Genotypes analysis

**Fig.2:**
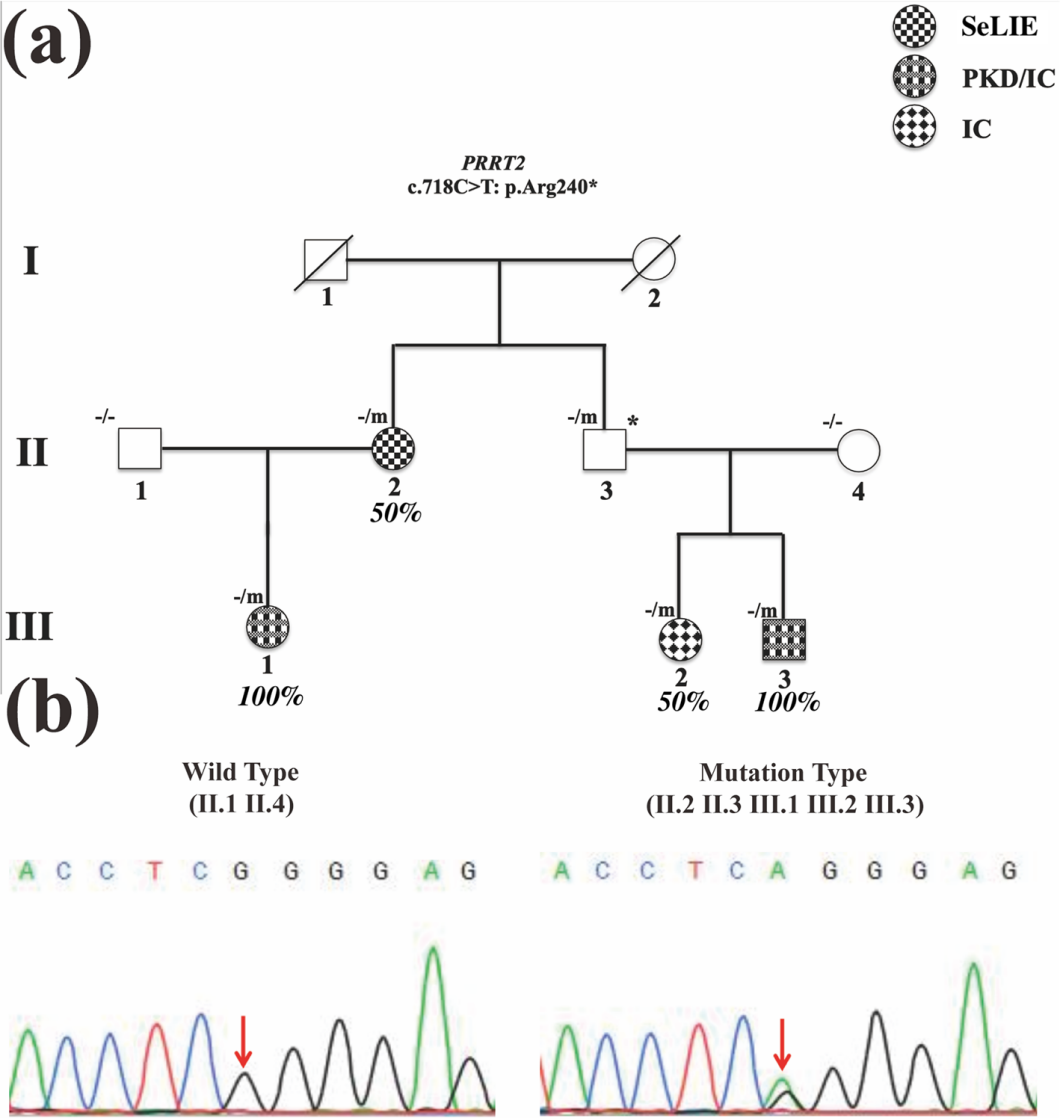
Phenotypic and genotypic data of the pedigree in this infantile convulsion and choreoathetosis syndrome family. **a** Familial pedigree. All unaffected family members were represented by white circles/squares, but one unaffected individual who carried *PRRT2* variant, c.718C > T: p.Arg240*, (II.3) was donated with an asterisk (*), which was recognized as an obligate carrier. All affected family members having different phenotypes were represented by different colored circles/squares, respectively. **b** Representative electropherograms of sanger sequencing were represented for all family members who underwent whole exome sequencing. Mutational locus was indicated by a red arrow. Note: -, wild type allele; m, mutation type allele; 50%, affected family members only presenting seizure symptoms without paroxysmal choreoathetosis symptoms, and can be diagnosed with SeLIE or IC; 100%, affected family members presenting seizure symptoms with paroxysmal choreoathetosis symptoms, and can be diagnosed with PKD/IC; IC, infantile convulsion; SeLIE, self-limited infantile epilepsy; PKD/IC, infantile convulsion with paroxysmal kinesigenic dyskinesia

The family-based whole-exome sequencing (WES) was performed in this infantile convulsion and choreoathetosis syndrome family, including all 4 affected available individuals (II.2, III.1, III.2, and III.3) and another 3 unaffected available individuals (II.1, II.3 and II.4), and a heterozygous *PRRT2* nonsense variant (NM_145239.3: c.718C > T, p.Arg240*) was identified. Sanger sequencing validated that this nonsense variant occurred in all 4 affected family members (II.2, III.1, III.2, and III.3) and 1 unaffected individual (II.3). No such variant was detected in 2 unaffected individual (II.1 and II.4) (Fig. 2a and b).

Considering *PRRT2* gene is a loss-of-function-related gene [13], the identified nonsense variant (p.Arg240*) create a premature translational stop signal in *PRRT2* gene and is expected to result in an absent or disrupted protein product; thus it should be regarded as a confirmed deleterious variant (PVS1). Moreover, this variant showed a very low minor allele frequency in the gnomAD database (0.000003125 in gnomAD v4.1.0 and 0.000004207 in gnomAD v2.1.1) (PM2_supporting). Additionally, this *PRRT2* variant (p.Arg240*) was previously reported in families with infantile convulsion and choreoathetosis syndrome [13–15] (PS4_moderate). According to American College of Medical Genetics variant classification system, this *PRRT2* variant could be classified as pathogenic variant (PVS1 + PM2_supporting + PS4_ moderate).

Consequently, those above phenotypic and genotypic findings of this family support that the diagnosis of *PRRT2*-related IC with developmental impairment and SeLIE could be made for the *pedigree* III.2 and *pedigree* II.2, respectively; meanwhile the diagnosis of *PRRT2*-related PKD/IC could be made for the *pedigree* III.3 and *pedigree* III.1. Moreover, according to the phenotypic and genotypic information of *pedigree* II.3, he could be defined as an obligate carrier (*i.e.*, an individual who is phenotypically unaffected but definitely has a pathogenic variant and passes it to his/her offspring in the basis of family history), and there was incomplete penetrance of *PRRT2* in this family, which may explain the multiple phenotypic features in this Chinese family with *PRRT2*-related syndrome.

## Discussion and conclusions

### Discussion

With the rapid development and popularization of WES technology, genetic causes are being identified more frequently than before in many unexplained neurological syndromes or disorders [16–18], including infantile convulsion and choreoathetosis syndrome. Since Chen et al. identified variants in *PRRT2* at 16p11.2 in eight Chinese families with infantile convulsion and choreoathetosis syndrome using WES data in 2011 [19], more than 330 families worldwide with *PRRT2* variants and infantile convulsion and choreoathetosis syndrome have been reported; *PRRT2* gene has been confirmed to be responsible for causing infantile convulsion and choreoathetosis. Nonetheless, another familial neurological syndrome, termed Rolandic epilepsy with paroxysmal exercise-induced dystonia and writer’s cramp (EPRPC, OMIM#608,105), which is caused by variants in *TBC1D24* gene, also shares overlapping clinical features with infantile convulsion and choreoathetosis, and shows phenotypic similarities to *PRRT2*-related syndrome [20]. Therefore, we should be cautious about other causes of infantile convulsion and choreoathetosis, like EPRPC, and cannot regard all patients exhibiting infantile convulsion and choreoathetosis symptoms as having *PRRT2*-related syndrome in clinical practice. *PRRT2* gene contains four exons and encodes PRRT2 protein that contains 340 amino acids and is predicted to have two transmembrane domains [21]. The PRRT2 protein is a neuro-specific protein highly expressed at the neuronal axons and presynaptic areas of cerebral cortex, basal ganglia, and cerebellum, involving in regulation of intrinsic excitability and neuromuscular transmitter release [19]. Previous studies have revealed that variants in *PRRT2* show incomplete penetrance with variable expressivity [22–25], which may explain the multiple phenotypic features in patients with this syndrome. In the present study, we identified a *PRRT2* nonsense variant (NM_145239.3: c.718C > T, p.Arg240*) that is resided in the proline-rich domain and conceivably leads to a truncation of the PRRT2 protein. The familial cases from Labate et al., Lee et al. and Cloarec et al. studies based on Caucasian populations carried the same amino acid alterations as our cases (p.ARG240*) [13–15]. Compared with those reported families, our cases showed more variable and heterogeneous phenotypic features. The reasons for such inconsistency phenotypic features of the same *PRRT2* variant could be as follows: (1) The various expressions of *PRRT2* in different generations may cause high heterogeneity; epistatic effects of other genes may affect the penetrance of *PRRT2* variants, leading to incomplete *PRRT2* penetrance and unpredictable haploinsufficiency phenotypes in our cases among different familial generations [26]. We thus cannot exclude the possibility that same variants in *PRRT2* may cause different phenotypes in them. (2) Moreover, the estimated penetrance of *PRRT2* variants was quite different between Asian variants carriers and Caucasian ones as previous studies reported [27]. Specifically, penetrance was higher in Asian cases carried *PRRT2* variants, including truncated and non-truncated variants, than in Caucasian ones. Thus, it is reasonable to speculate that an Asian descendant with a pathogenic *PRRT2* variant is more likely to have high *PRRT2* penetrance with more variable phenotypes than a Caucasian one with the same variant. (3) Other genetic differences, like epigenetic modification or single nucleotide polymorphism, and environmental differences may also affect *PRRT2* penetrance; further researches are necessary to test those effects [28]. Meanwhile, more attention should be paid by genetic clinicians to the interpretation of this identified variant, c.718C > T (p.Arg240*).

On the other hand, developmental impairment including GDD and severe GR, identified from the *pedigree* III.2, may be a novel phenotypic trait related to the c.718C > T (p.Arg240*) *PRRT2* mutation. The c.718C > T (p.Arg240*) nonsense variant located within the segment of exon 2 that encoded the proline-rich domain of PRRT2. This variant thus can lead to a truncated protein with incomplete proline-rich domain, and impair the subcellular localization, further leading to dysfunctions of its membrane targeting and transmembrane effects [13–15]. The latter function is predicted to influence the construction of some specific types of ion channels [19]. Meanwhile, previous reports has revealed that truncated PRRT2 protein, especially those with incomplete proline-rich domain, is more Likely to lose its ability to interact with synaptosomal-associated protein 25, a plasma membrane-bound protein highly expressed in presynaptic domains and involved in the regulation of neurotransmitters release [1]. We therefore speculate that the impaired functions related to construction of specific types of ion channels and neurotransmitters release caused by c.718C > T (p.Arg240*) may give rise to neurodevelopmental impairment and following neuromodulated growth impairment (such as inhibiting the secretion of growth hormone) underlying the *PRRT2*-associated IC condition. Interestingly, previous studies have found two infantile convulsion and choreoathetosis syndrome patients carried the same variant, c.718C > T (p.Arg240*), could present other rare PRRT2-associated phenotypes, including migraine without aura [15] and febrile seizures with probable sudden unexpected death in epilepsy [14], indicating that the high phenotypic variability in patients carried this *PRRT2* variant; the *pedigree* III.2 in our study showing uncontrolled multiple seizure types with developmental impairment could further provide clinical evidences to support this speculation, which warrants further research attention.

Moreover, It should be noted that with the progression of epileptic condition, there was aggravation in the severity of GR in *pedigree* III.2 who did not receive any effective antiepileptic medications. This finding may indicate that the phenotype of developmental impairment in our patient may be not only related to *PRRT2* variant, but also have close links to the uncontrolled epileptic condition, which implies the pathogenic *PRRT2* variant responsible for the epilepsy itself may also lead to developmental disorders, like other genetic epileptic and developmental encephalopathies such as *SCN1A*-related epileptic encephalopathy. We therefore posit that the prognosis of children with *PRRT2*-related seizures might not always be benign or self-limited; children having *PRRT2*-related seizures, especially those who present multiple epileptic forms, still need timely and effective AEDs to control epileptic attacks in case to prevent potential developmental impairment caused by recurrent uncontrolled seizures.

### Conclusions

In summary, our current case study provides some new phenotypic clues to the developmental impairment with growth restriction caused by a pathogenic heterozygous *PRRT2* variant (NM_145239.3: c.718C > T, p.Arg240*), which may not only enlarge the clinical spectrum related to *PRRT2*-related syndrome, but also underscore the importance of assessing developmental status and controlling epileptic attacks for preventing potential developmental impairment in patients with this syndrome.

## Supplementary Information

Supplementary Material 1.

Supplementary Material 2.

Supplementary Material 3.

Supplementary Material 4.

Supplementary Material 5.

Supplementary Material 6.

Supplementary Material 7.

Supplementary Material 8.

## Data Availability

No datasets were generated or analysed during the current study.

## Abbreviations

AEDs: Antiepileptic drugs
CBZ: Carbamazepine
DQ: Developmental quotient
EEG: Electroencephalogram
GDD: Global developmental delay
GR: Growth retardation
GTCSs: Generalized tonic–clonic seizures
IC: Infantile convulsion
LEV: Levetiracetam
MRI: Magnetic resonance imaging
PKD: Paroxysmal kinesigenic dyskinesia
PKD/IC: Infantile convulsion with paroxysmal kinesigenic dyskinesia
PRRT2: Proline-rich transmembrane protein 2
SeLIE: Self-limited infantile epilepsy
WES: Whole-exome sequencing
