# Supplementary material for: Multiple phenotypic traits including developmental impairment in a Chinese family with infantile convulsion and choreoathetosis syndrome: a case study expanding the clinical spectrum of prrt2-related syndrome
Source: BMC Pediatr. 2025 Oct 6;25:769. doi: 10.1186/s12887-025-06180-9 (PMC12502556; doi:10.1186/s12887-025-06180-9)
Supplement: Supplementary file 1 — Supplementary Material 1. [file 12887_2025_6180_MOESM1_ESM.docx]

**Supplementary Videos Legends**

**Supplementary video 1:** Video recording an afebrile generalized tonic-clonic seizure attack in the *pedigree* III.2 at the age of 5 months.

**Supplementary video 2:** Video recording one focal seizure with impaired awareness in the *pedigree* III.2 at the age of 1 year and 3 months.

**Supplementary video 3:** Video demonstrating a typical afebrile generalized tonic-clonic seizure attack in the *pedigree* III.3 at the age of 4 months.

**Supplementary video 4:** Video revealing a series of choreiform limb movements mixing with left upper limb ballism in the *pedigree* III.3 at the age of 4 months.

**Supplementary video 5:** First-person perspective video capturing a typical paroxysmal kinesigenic dyskinesia of left upper limb mixing with left lower limb triggered by suddenly flipping the palm in the *pedigree* III.1 at the age of 17 years.
