# Supplementary material for: Multiple phenotypic traits including developmental impairment in a Chinese family with infantile convulsion and choreoathetosis syndrome: a case study expanding the clinical spectrum of prrt2-related syndrome
Source: BMC Pediatr. 2025 Oct 6;25:769. doi: 10.1186/s12887-025-06180-9 (PMC12502556; doi:10.1186/s12887-025-06180-9)
Supplement: Supplementary file 2 — Supplementary Material 2. [file 12887_2025_6180_MOESM2_ESM.docx]

**Materials and Methods**

**Family collection and ethical compliance**

A Chinese Han family with infantile convulsion and choreoathetosis syndrome including 4 affected individuals and 3 related unaffected individuals was enrolled and investigated in this study at Sun Yat-sen Memorial Hospital of Sun Yat-sen University. Phenotypic data of all enrolled family members were obtained and collected via face-to-face interviews, mainly including cardinal clinical manifestations (such as onset/remission age of seizures and choreoathetosis symptoms, duration/frequency and phenomenology features of attacks or a video of one typical attack episode if available), cardinal information of auxiliary examinations (such as neuro-electrophysiology and neuroimaging tests when available) and medical history information (such as present/past illness history, family history, personal history and so on). The International League Against Epilepsy criteria were applied to diagnosis seizures and epilepsy. Clinical diagnostic criteria for global development delay (GDD) were based on the Diagnostic and Statistical Manual of Mental Disorders, 5^th^ Edition (DSM-V). Informed written consents for genetic investigation and publication were obtained from all involved family members or their guardians. Informed written permissions of using individuals’ pictures or video materials containing detailed seizure/attack features (including facial and limbic symptoms) for publication were obtained from all involved family members or their guardians. All procedures of the study were done in agreement with the Declaration of Helsinki, and approved by the Ethics Committee of Sun Yat-sen Memorial Hospital (Approval Number: SYSKY-2024-384-01)

**Genetic investigation**

Peripheral blood samples were obtained from these 7 enrolled family members. Genomic DNA was extracted and purified from obtained blood samples by applying QiAamp blood mini kit (Qiagen, Shanghai, China) according to the protocols of manufacturer. Extracted qualified DNAs were then processed with whole-exome sequencing (WES). In short, we applied Illumina TruSeq exome kit (Illumina, San Diego, USA) to establish DNA libraries according to the instructions of manufacturer. The Illumina Novaseq 6000 (Illumina, San Diego, USA) was further used to perform sequencing based on the standard protocols of manufacturer, and a total of ~ 10-GB exome data per individual were generated. Exome data analysis was performed using commercial pipeline developed by GeneRanger (Xunyin Biotech, Shanghai, China). Reads alignment, indel regions realignment, base qualities recalibration, variant capture, and calling/transformation were processed using Burrows-Wheeler aligner, Picard tool and Genome Analysis ToolKit. Based on the standard criteria reported by the Genome Aggregation Database (gnomAD), variant filtering was set on coverage depth ≥ 10 with minor allele frequency ＜ 0.05%. Assessment and interpretation of pathogenicity of detected variant was based on the variant classification system developed by American College of Medical Genetics Validation of WES-identified variant was performed using Sanger sequencing according to the standard protocols. The primer sequence for Sanger sequencing of *PRRT2* variant identified in this research was demonstrated as follows: Forward, AAGAGAATGGGGCAGTGGTG; Reverse, TAAGCGAAGGCCACGATGTT
