# Supplementary material for: Multiple phenotypic traits including developmental impairment in a Chinese family with infantile convulsion and choreoathetosis syndrome: a case study expanding the clinical spectrum of prrt2-related syndrome
Source: BMC Pediatr. 2025 Oct 6;25:769. doi: 10.1186/s12887-025-06180-9 (PMC12502556; doi:10.1186/s12887-025-06180-9)
Supplement: Supplementary file 3 — Supplementary Material 3. [file 12887_2025_6180_MOESM3_ESM.pdf]

CARE
case report guidelines

CARE Checklist of information to include when writing a case report

(cc) BY-NC-ND

Topic	Item	Checklist item description	Reported on Line
Title	1	The diagnosis or intervention of primary focus followed by the words "case report"	Page 1 Line 2-6
Key Words	2	2 to 5 key words that identify diagnoses or interventions in this case report, including "case report"	Page 2 Line 30-31
Abstract (no references)	3a	Introduction: What is unique about this case and what does it add to the scientific literature?	Page 2 Line 21-27
	3b	Main symptoms and/or important clinical findings	Page 2 Line 14-20
	3c	The main diagnoses, therapeutic interventions, and outcomes	Page 2 Line 12-14
	3d	Conclusion—What is the main "take-away" lesson(s) from this case?	Page 2 Line 24-27
Introduction	4	One or two paragraphs summarizing why this case is unique (**may include references**)	Page 3 Line 30-Page 4 Line 3
Patient Information	5a	De-identified patient specific information.	NA
	5b	Primary concerns and symptoms of the patient.	Page 4 Line 7-Page 5 Line 13
	5c	Medical, family, and psycho-social history including relevant genetic information	Page 6 Line 18-25
	5d	Relevant past interventions with outcomes	Page 4 Line 16-21
Clinical Findings	6	Describe significant physical examination (PE) and important clinical findings.	Page 5 Line 3-13
Timeline	7	Historical and current information from this episode of care organized as a timeline	Page 5 Line 3-13
Diagnostic Assessment	8a	Diagnostic testing (such as PE, laboratory testing, imaging, surveys).	Page 6 Line 18-25
	8b	Diagnostic challenges (such as access to testing, financial, or cultural)	NA
	8c	Diagnosis (including other diagnoses considered)	Page 7 Line 3-12
	8d	Prognosis (such as staging in oncology) where applicable	NA
Therapeutic Intervention	9a	Types of therapeutic intervention (such as pharmacologic, surgical, preventive, self-care)	NA
	9b	Administration of therapeutic intervention (such as dosage, strength, duration)	NA
	9c	Changes in therapeutic intervention (with rationale)	NA
Follow-up and Outcomes	10a	Clinician and patient-assessed outcomes (if available)	NA
	10b	Important follow-up diagnostic and other test results	NA
	10c	Intervention adherence and tolerability (How was this assessed?)	NA
	10d	Adverse and unanticipated events	NA
Discussion	11a	A scientific discussion of the strengths AND limitations associated with this case report	Page 7 Line 17-Page 9 Line 31
	11b	Discussion of the relevant medical literature **with references**.	Page 7 Line 17-Page 9 Line 18
	11c	The scientific rationale for any conclusions (including assessment of possible causes)	Page 9 Line 19-31
	11d	The primary "take-away" lessons of this case report (without references) in a one paragraph conclusion	Page 10 Line 1-6
Patient Perspective	12	The patient should share their perspective in one to two paragraphs on the treatment(s) they received	NA
Informed Consent	13	Did the patient give informed consent? Please provide if requested	Yes ☑ No ☐
